# Classification of airborne multispectral imagery to quantify common vole impacts on an agricultural field

**DOI:** 10.1002/ps.6857

**Published:** 2022-03-16

**Authors:** Javier Plaza, Nilda Sánchez, Carmen García‐Ariza, Rodrigo Pérez‐Sánchez, Francisco Charfolé, Constantino Caminero‐Saldaña

**Affiliations:** ^1^ Plant Production Group. Faculty of Environmental and Agricultural Sciences University of Salamanca Salamanca Spain; ^2^ Department of Cartographic and Land Engineering University of Salamanca Ávila Spain; ^3^ Pest Area. Technological Agricultural Institute of Castilla y León (ITACyL) Valladolid Spain

**Keywords:** alfalfa, classification, *Microtus arvalis* Pallas, multispectral, NDVI, UAS

## Abstract

**BACKGROUND:**

The common vole (*Microtus arvalis*) is a very destructive agricultural pest. Particularly in Europe, its monitoring is essential not only for adequate management and outbreak forecasting, but also for accurately determining the vole's impact on affected fields. In this study, several alternatives for estimating the damage to alfalfa fields by voles through unmanned vehicle systems (UASs) and multispectral cameras are presented. Currently, both the farmers and agencies involved in the integrated pest management (IPM) programs of voles do not have sufficiently precise methods for accurate assessments of the real impact to crops.

**RESULTS:**

Overall, the four multispectral classification methods presented showed similar performances. However, the normalized difference vegetation index (NDVI)‐based segmentation exhibited the most accurate and reliable appraisal of the affected areas. Nevertheless, it must be noted that the simplest method, which was based on an automatic classification, provided results similar to those obtained by more complex methods. In addition, a significant direct relationship was found between the number of active burrows and damage to the alfalfa canopy.

**CONCLUSION:**

Unmanned vehicle systems, combined with multispectral imagery classification, are an effective and easily transferable methodology for the assessment and monitoring of common vole damage to agricultural plots. This combination of methods facilitates decision‐making processes for IPM control strategies against this pest. © 2022 The Authors. *Pest Management Science* published by John Wiley & Sons Ltd on behalf of Society of Chemical Industry.

## Graphical abstract

Common vole damages in the alfalfa canopy obtained throughout UAS multispectral imagery classification methods were significantly related to the number of active vole burrows. Among the four remote methodologies considered, NDVI‐based segmentation exhibits the most accurate and reliable appraisal of the affected areas.

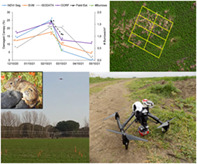

## 1 INTRODUCTION

European agricultural landscapes host numerous species of small mammals (mainly rodents), some of which are considered to be highly destructive agricultural pests.^1^ A well‐known and highly prevalent pest is the common vole (*Microtus arvalis* Pallas 1778), hereafter referred to as the vole. It is a semifossorial microtinid that primarily inhabits agricultural landscapes such as meadows, field margins and alfalfa plots.^2^ However, during periods of population outbreak, voles disperse, invade and colonize other suboptimal habitats, including areas cultivated with a wide range of crops (e.g. cereals, legumes, rapeseed, sunflower).^2^, ^3^ In some Mediterranean regions, such as the farming lands of the west‐central part of the Iberian Peninsula (Spain), voles cause significant damage as a consequence of their increased prevalence.^4^

However, the population and prevalence increase are caused not only by the inherent behavior of the voles, but also by human modification of their natural environment.^5^, ^6^ An anthropogenic modification to a natural environment often is a precursor to the emergence and propagation of zoonotic diseases such as tularemia, caused by *Francisella tularensis* bacterium,^7^ and population explosions among pests that can wreak havoc on agricultural production. Specifically, voles undergo a recurring demographic phenomena every 2–5 years, during which they multiply very quickly and attain densities of >2000 individuals ha^−1^ in extreme cases.^1^, ^6^ This phenomenon leads to the biological invasion and colonization of all spaces, including cultivated plots.^8^ Indeed, extraordinary explosions of vole populations have occurred in recent decades in the agricultural areas of the Castilla y León region (Spain). An instance occurred in 2007 (between 3 and 4 million ha of agricultural landscapes were invaded), when overabundant vole populations caused the highest loss of cereals, potatoes and vineyards observed in over a decade in this region. Mitigation costs were estimated at €15 million.^4^, ^8^ This led to mandatory and systematic monitoring of vole populations and their incidences of damage. Monitoring is crucial to optimize the management and to predict an outbreak of voles, because monitoring not only allows for assessments of the presence of voles and the resulting damage in a specific plot at a given time, but also allows for the establishment of preventive actions to effectively reduce the spread of a plague.

Currently, vole monitoring is performed through laborious and time‐consuming fieldwork. Trapping methods are very effective field procedures for estimating vole populations, although they require significant human and material resources. For this reason, they are usually replaced by indirect methods, based on detecting signs of vole activity (i.e. droppings, active burrows and vegetation clippings), which are easier and faster.^9^ These estimations of activity usually are linked to reduced vegetation cover.^10^ Quantification of the reduction in vegetation cover is based on a sampling hierarchy, which often involves subjective biases emanating from the criteria used by the technician performing the sampling procedure.

In this context, photography, video technologies and remote sensing imagery have emerged as very useful tools for monitoring rodent populations.^11^, ^12^ For instance, satellite images have been used to monitor agricultural damage caused by rats^13^, ^14^ and large gerbil burrows,^15^ and to predict a microhabitat's suitability for endangered rodent species.^16^ Unfortunately, satellite images have coarse spatial resolutions. However, other low‐altitude remote sensing methods, such as unmanned aerial systems (UASs), commonly known as drones, are currently available to solve this resolution problem. Recently, drones have been used widely in landscape ecology studies because of their high spatial resolutions, low cost and easy operability.^17^ Presently, there is scientific literature related to the use of drones and red/green/blue (RGB)‐based imagery for rodent infestation assessment and monitoring. In regards to rodent populations, two main areas have been explored in the literature: (1) survey and recognition of rodent infestation^11^, ^18^, ^19^ and (2) automatic identification of rodent evidence, such as burrows, by following a feature‐extraction schema.^12^, ^20^ A third group of investigations explores objective assessments of the damaged areas caused by the rodents, but in this case, the research relies on remote sensing, mainly by the use of Moderate Resolution Imaging Spectroradiometer (MODIS) imagery.^21^ Currently, very little scientific research exists on the combined use of UAS and multispectral imagery for assessments of rodents' impact on agricultural fields, and more specifically in relation to the common vole. However, identifying the crop damage resulting from rodent infestations is vital for agricultural managers. The fine scale of the crop canopy damage caused by the voles makes necessary an evaluation at plot level, typically with pixel resolution of few centimeters. This suggests the use of very high‐resolution imagery, which can be gathered by close‐range flights done by UAS. Unmanned aerial system imagery enables estimations of the proportion of affected vegetation cover, plant foliar area or a segmentation of the soil or damaged areas, among other parameters.^22^ Multispectral sensors are particularly useful in these estimations, owing to their inclusion of infrared bands, which are sensitive to plant vigor.^23^ The most common method to assess the percentage of vegetation loss and damage to the canopy, regardless of the cause of the loss, is through classification methods applied to the imagery inputs. These methods include the ‘unsupervised’ clustering initiated in the late 1970s, to the current ‘deep learning’‐based classifications.^24^ There is an extensive body of scientific literature regarding developing methods such as subpixel, knowledge‐based, contextual‐based, object‐based classification analysis and hybrid approaches.^25^ However, little effort has been devoted to distinguishing pest‐damaged areas from healthy vegetation at a fine scale. For example, classification methods can help differentiate fully vegetated areas from ‘bald’ areas created by voles^20^ and also identify intermediate stages of damage. In pest damage evaluations, different classification approaches have been used, such as vegetation index thresholds,^21^ automatic classification methods^26^ and supervised classifications based on machine learning, and either pixel‐oriented (each pixel is classified independently)^27^ or object‐oriented methods (all pixels within defined objects are included to define spectral behavior through an iterative classification process).^28^ Imagery segmentations of damaged areas from UASs also have been used in the diagnoses of precision applications of control measures by drones.^29^ Considering the above, through our research, we developed a UAS‐based alternative to accurately evaluate the impact of the common vole in an agricultural field. Our principal motivation was to assist farmers and pest managers who currently do not have methods to accurately determine vole impact on their crop fields. The secondary objective was to test different image classification methods as well as to compare them with traditional field observations of vole impacts. To do so, both the fine‐scale and spectral capabilities of an airborne multispectral sensor to monitor the impact of a common vole plague on crop fields were explored. In particular, an alfalfa field in the center of the Iberian Peninsula was monitored during 2020–2021 to assess the potential of this methodology.

## 2 MATERIALS AND METHODS

### 2.1 Study area

**Figure 1:**
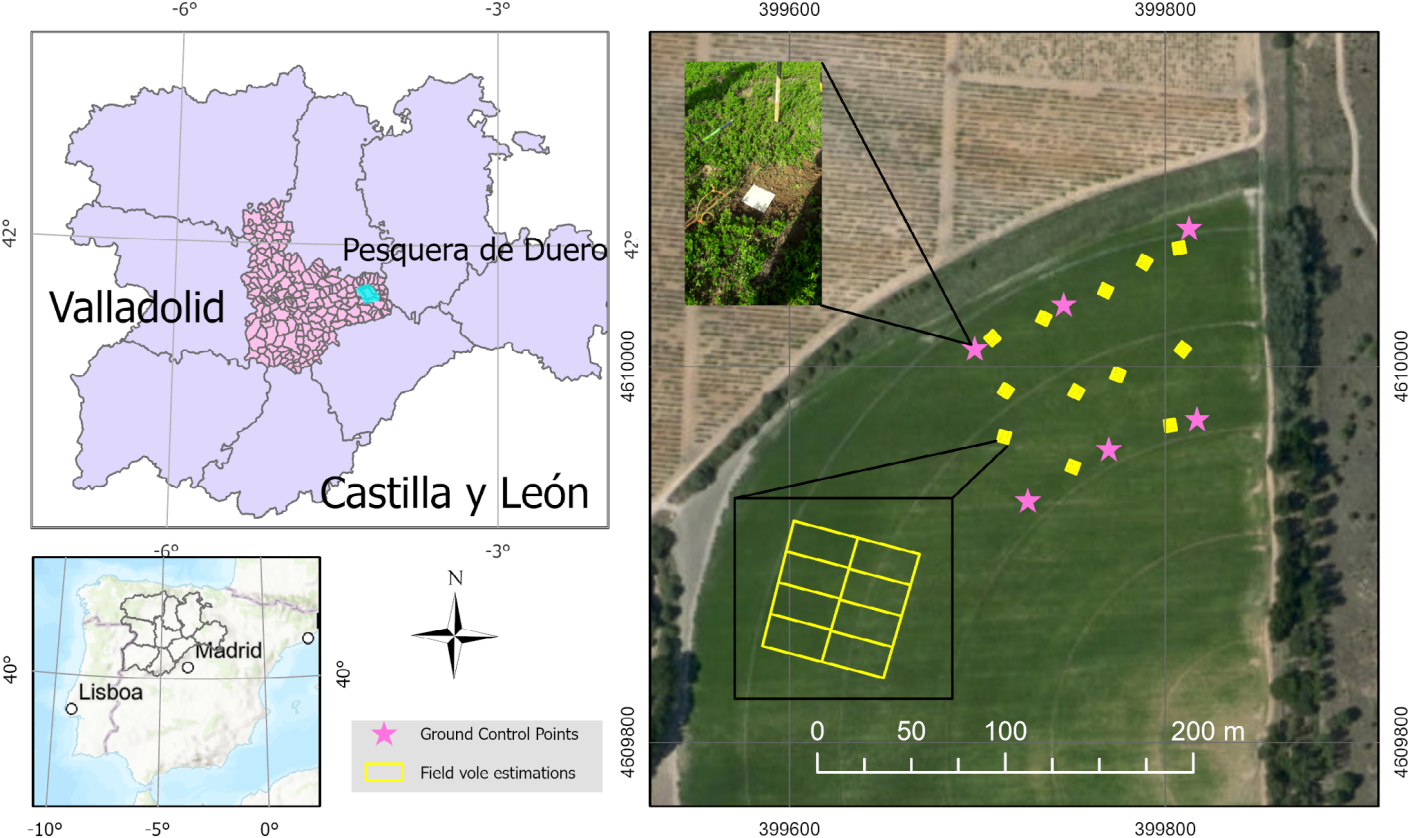
Map indicating the study area, including the squares where the field estimations took place (in yellow) and the GPS ground control points (in pink).

This study was carried out in an irrigated alfalfa (*Medicago sativa* L.) field located in the province of Valladolid (41°38′01”N‐4°12′14”W, 741 m above sea level), which belongs to the region of Castilla y León, in the northern center of the Iberian Peninsula (Fig. 1). The study was conducted from December 2020 to May 2021 between two alfalfa harvests (14 September 2020 and 21 May 2021). Thus, during the entire study period, the alfalfa remained unharvested. This region has a Mediterranean‐continental climate that is characterized by cold winters and hot summers, with a very short spring and autumn and a frost period from October to April. Temperatures are considered extreme, exceeding 35 °C in summer and − 12 °C in winter (Spanish State Meteorological Agency, AEMET, http://www.aemet.es/en/). Annual rainfall in this region is remarkably scarce and irregular, ranging from 300 to 400 mm.

A monitoring and surveillance program carried out by the regional government has detected vole population increases since the summer of 2020. Moreover, owing to its location and management system, this specific zone is representative of the irrigated alfalfa plots in the area.

### 2.2 Data acquisition and pretreatment

#### 2.2.1 Field data

Four drone flights were conducted during the study period (18 December 2020, 19 February 2021, 10 March 2021 and 4 May 2021). Simultaneously, field estimations were taken as described in the following sections.

#### 2.2.2 GPS collection

The GPS equipment was a 1200 Leica GPS receiver (Leica Geosystems, Heerbrugg, Switzerland) composed of a Leica Smartover ATX 1200 GNSS 1200 GG & GPRS dual‐frequency modem (compatible with GPS and GLONASS signals), a Leica GX1220 GPS and GLONASS dual‐frequency geodetic receiver for fixed or static observations, a standard antenna for a GX1220 receiver, a Satelline radio modem and a mobile phone SIM card. Real‐time kinematic (RTK) observations were recorded using the ITACyL network services, guaranteeing a final geolocation error of <2 cm. This system allowed for accurate geolocation and orthomosaiking of UAS images. To produce these images, six ground control points (GCPs) (i.e. target plates) were strategically placed in the field (Fig. 1) and were recorded with GPS. In addition, GPS measurements were recorded in the damaged areas to use them as ground truths to validate the results of the four classification methods (*n* = 111 locations throughout the flight area). The validation points comprised burrows, tracks and bare stands. These field measurements took place on dates 18 December 2020 (*n* = 58 validation points), 19 February 2021 (*n* = 36) and 10 March 2021 (*n* = 17), excluding the last flight in May, because there were no visibly affected areas at that time. It should be noted that there were fewer validation points in May because there was less damaged surface at this date, as will be shown in the Results section.

#### 2.2.3 Vole field monitoring

**Figure 2:**
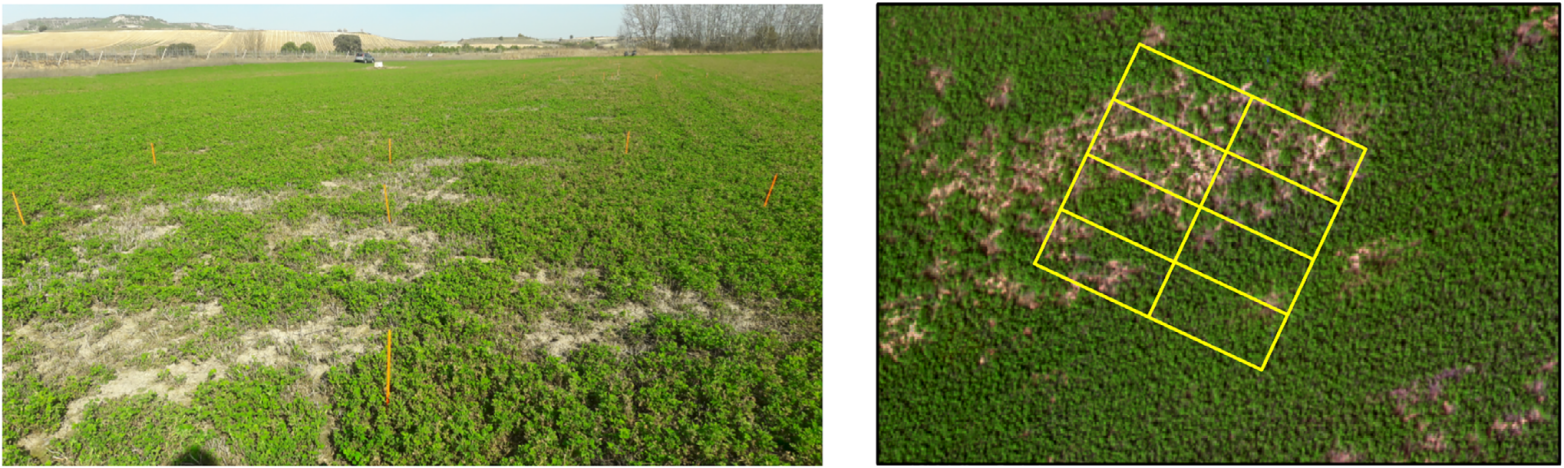
Squared plots of the vole field estimations at the field scale (left) and by drone image (right). Date acquisition 19 February 2021.

A total of 12 6 × 6 m^2^ plots were field‐designed and inspected in this work. Each of them was divided into eight sample units of 3 × 1.5 m^2^ (Fig. 2), which were established as experimental units for further analyses. The squares were selected after a careful inspection of the plot. They were distributed regularly within a maximum area of 1 ha (fitting the flight extents), and separated by 20 m from the nearest square and from the closest field margin.

In each sample unit, the degree of active vole colonization and canopy damage was assessed. To ensure that the selected colonies within the squares were active, two methods were used. The first method included the detection of the vole activity linked to the colonies (fresh droppings, recent digging and/or vegetation clipping).^30^ The second method included burrow renewal,^31^ in which all the burrow mouths were lightly covered with soil so that voles, if present, could easily reopen them. After 24 h, the number of reopened burrows was counted. Vole activity was estimated by counting the number of burrow entrances (hereafter ‘burrows’). In this study, all of the observed colonies showed activity according to the two abovementioned methods.

In order to evaluate the magnitude of damage to vegetation cover caused by the voles found in each experimental plot, a visual scale from 0 (total affectation with absence of plants) to 6 (no affectation, no absence of plants) was used (Table 1). This visual assessment was always conducted by the same technician.

**Table 1:** Scale used to assess the effect of common vole infestation on the vegetation cover

Code	Estimation of affected area (%)
0	100
1	80–100
2	60–80
3	40–80
4	20–40
5	0–20
6	0

These field estimations took place on the dates 19 February 2021 and 10 March 2021, on the same day, immediately after the flights of February and March.

#### 2.2.4 Drone flights

Four flights were conducted between December 2020 and May 2021 at an altitude of 26 m, resulting in four maps at a spatial resolution of 2 cm. Each flight covered an area of ≈1 ha within the alfalfa field.

A Micasense RedEdge‐M camera (AgEagle Sensor Systems Inc., d/b/a MicaSense, Wichita, KS, USA) was mounted on a DJI‐Inspire‐1 drone (SZ DJI Technology Co., Ltd., Shenzhen, China). This camera has five‐band images in the visible–near infrared (VNIR) space, namely, blue, green, red, red‐edge and near‐infrared wavelengths. The onboard Micasense equipment also included a GPS receiver and a downwelling light sensor (DLS) to account for the illumination conditions during each acquisition, which were taken around noon (11:00–13:00 UTC) to avoid shadows. In addition, to convert the raw values stored in each band into absolute reflectance, several images of a calibrated reflectance panel were taken before and after each flight. This procedure is designed to obtain a fair comparison between the derived image products of each date that were used for the analysis.

The flight mission design and image processing were performed using pix4d capture and pix4d mapper (Pix4D P.A., Prilly, Switzerland), respectively. The latter allowed geometric correction, radiometric calibration and orthomosaicing of the absolute reflectance maps, following a standard workflow described in detail by Plaza *et al*. (2021).^32^

### 2.3 Estimation of the affected area: classifications

Voles are able to invade irrigated crops year‐round, especially pluriannual crops such as alfalfa, which provide them with continuous food and shelter.^2^ In fact, voles can consume between 1.6% and 45.8% of the annual alfalfa production; consumption can reach >80% in outbreak events.^8^ In addition, given that alfalfa is harvested several times each year, the losses are cumulative. Voles remove and eat both the aerial parts of the plants and their roots, creating clearance areas without vegetation around the burrows (Fig. 2). These bare soil stands account for crop‐damaged areas, and also comprise burrows, paths and turned‐over soil. In the absence of other biotic or abiotic causes, such as water or nutritional deficiencies or any other plague/disease, we considered vole activity to be the sole cause of alfalfa decay and loss.

All image analyses were performed in arcgis pro 2.6 (Environmental Systems Research Institute, Redlands, CA, USA). Because the classification was intended to separate only two categories (i.e. ‘damaged’ *versus* ‘nondamaged’ areas – three simple and conventional methods of image classification were proposed, all categorized as ‘pixel‐oriented’ methods. The high spatial resolution of the UAS imagery (without mixed spectral characteristics) also indicated there was no need for subpixel classifications.

The simplest method was NDVI segmentation, a widely used method to partition the landscape into bare soil and vegetation.^33^ The procedure of Wilschut *et al*.^15^ and Xu *et al*.^21^ was adapted for the task. First, 20 points of the damaged areas within the bare stands scattered throughout the image were visually selected and used as training points. Second, buffers of a radius of 2 pixels (4 cm) were applied at each point, assuming that the minimum damaged area slightly exceeded the width of a burrow‐mouth size, which ranged between 1.9 and 4.5 cm,^34^ due to the absence of plants and the soil accumulation around it. Therefore, an area of ≈7–8 cm in diameter was considered the minimum affected unit.

The maximum, minimum, median and quartiles of the NDVI values at each buffer were extracted. After several tests, the criterion of the maximum NDVI (averaged for all buffers) was selected as the ‘damage’ threshold. Therefore, all pixels with NDVI values below this threshold were considered damaged areas.

The second method is a supervised classification based on the Support Vector Machine (SVM) algorithm using the classification tool in the arcgis pro 2.6 platform.^35^ The five multispectral bands of the camera were used as the input for the classification. To train the model, the same visual selection of damaged areas was used as in the previous methods.

The third method is a pixel‐based automatic classification without training samples, using the ISO Cluster classifier available in arcgis pro 2.6 (Iterative Self‐Organization Data analysis, ISODATA),^36^ which assigns pixels to both classes based on their spectral characteristics. Because it is an unsupervised method, there is no need to train the damaged areas. For the supervised method, the five bands of Micasense Red_edge M were computed.

Alternatively, a more complex ‘object‐oriented’ method of classification was applied. The object‐based classification is similar to the pixel‐based classification, with the difference that all the pixels are combined in the objects and are classified together.^37^ Therefore, this method requires a previous step of grouping neighboring pixels, the so‐called segmentation, based on the integration of the spectral, shape and size characteristics of each group. In this case, we chose the values of 16, 15 and 10, respectively, to balance the importance of their spectral/geometric characteristics, as well as the super‐high spatial resolution of the image inputs. The second step consisted of creating training samples for the features resulting from the segmentation. Finally, the classification algorithm was applied. In this case, after several tests of the four available algorithms in arcgis, the random trees classifier^38^, ^39^ was selected for its slightly better performance.

An accuracy assessment of the four methods consisted of testing the coincidence of the resulting damaged areas in the image with those taken in the field as ground truths, which was overlaid on each classification and date. Overall accuracy was defined as the percentage of correctly classified damaged areas. By contrast, the true‐damaged areas observed in the field that were incorrectly assigned as healthy vegetation in the segmented maps were deemed as the percentage of ‘false negatives’ (100% minus the percentage of correct). No ‘false‐positives’ were assessed because only damaged areas were gathered with the GPS as the ground truth.

A final accuracy assessment of the resulting maps consisted of comparing the ranking of severity of the field estimations (estimated at the sub‐square level) with the percentages in the classified maps extracted for each sub‐square.

### 2.4 Statistical analysis

The relationship between the percentage of damaged canopy resulting from the classifications and field observations (number of burrows m^−2^) was assessed using a bivariate correlation process based on Pearson's linear correlation coefficient as the correlation indicator.^40^ A two‐tailed significance test was performed, flagging significant ($P\leq 0.05^{*}$) and highly significant ($P\leq 0.01^{**}$) correlations. These correlations were calculated using data collected from 96 sample units on 19 February 2021 and 10 March 2021 (n=192).

Significant differences among remote methodologies and field estimations were obtained using one‐way analysis of variance (ANOVA). The means and standard errors (SEs) were calculated for all variables. The statistical significance was assessed at a 95% confidence level (α=0.05) using Snedecor's *F* as the contrast statistic. For differentiation of homogeneous subsets, honestly significant difference (HSD) Tukey's test was used.^41^ For a better understanding of the results, and to avoid redundant information, the results of this analysis will be presented together with the graphical output of the comparison between the different methodologies.

Statistical processing was performed using the IBM‐SPSS statistics 26 software (IBM, Chicago, IL, USA).

## 3 RESULTS

### 3.1 Accuracy assessment of the four classification methods

The overall accuracy – the percentage of field damaged areas correctly identified in the classified maps (Table 2) – indicated that the NDVI segmentation was the most accurate method (overall accuracy >80% for all dates and <20% of false negative identifications), closely followed by the object‐oriented random forest classification (OORF) and then by the SVM supervised and ISODATA unsupervised classifications. In general, all the methodologies were similar in accuracy (>70% on average). Therefore, NDVI segmentation showed the best performance among the four methods. It is noteworthy, however, that the simplest method, the unsupervised ISODATA, performed in a similar way (overall accuracy 72.9%) to the more complex methods of supervised classification (72.4% for SVM and 80.2% for OORF) or NDVI segmentation (84.2%), both of which require training datasets and a higher level of knowledge.

**Table 2:** Accuracy assessment of the four classification methods

Flight date	NDVI segmentation	Supervised pixel‐based classification (SVM)	Unsupervised pixel‐based classification (ISODATA)	Supervised object‐based classification (OORF)
18 December 2020	84.0	80.0	76.0	92.0
19 February 2021	82.9	65.7	57.1	71.4
10 March 2021	85.7	71.4	85.7	77.1
Average	84.2	72.4	72.9	80.2

NDVI, normalized difference vegetation index; SVM, support vector machine; ISODATA, iterative self‐organizing data analysis; OORF, object‐oriented random forest.

### 3.2 Percentage of damage and accuracy assessment

**Figure 3:**
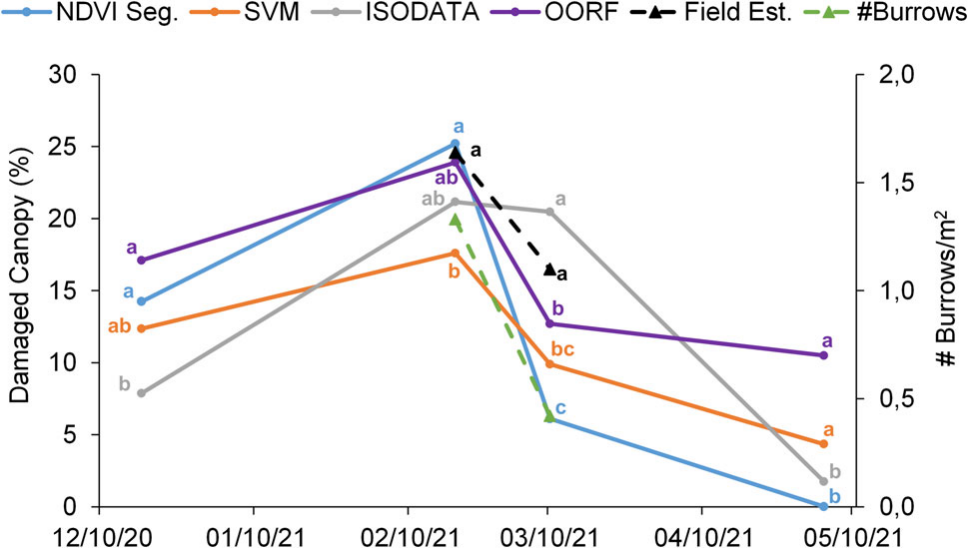
Damaged canopy and number of burrows simultaneously accounted for the field estimations. Lower case letters refer to the different homogeneous subsets resulting from the HSD‐Tukey analysis. NDVI, normalized difference vegetation index; SVM, support vector machine; ISODATA, iterative self‐organizing data analysis; OORF, object‐oriented random forest; Field Est., field estimations of damaged areas and #Burrows/m^2^: number of burrows m^−2^.

Figure 3 shows the damaged canopy (%) using the four different methods, together with the field estimations. The total number of burrows m^−2^ also was included.

The four classifications showed a similar trend, which was also corroborated by field estimations of the damaged areas and the number of burrows. In all cases, an increasingly damaged canopy was revealed from December to February, after which it drastically plummeted, albeit not as sharply as in the case of the ISODATA classification. This event was directly related to a lower presence and activity of voles in the study area, as evidenced by the reduction in the number of burrows m^−2^ from February (1.33 burrows m^−2^) to March (0.42 burrows m^−2^). Immediately after that period, the alfalfa gradually recovered from the damage until the end of the study period, and the affection was negligible.

The NDVI segmentation indicated that the highest canopy damage occurred in December and February, finding no differences from the results obtained in the field measurements later on in the growing season. Conversely, it was the methodology that showed the lowest values for canopy damage to occur during March and May. Furthermore, the ISODATA classification registered the lowest percentage of damaged canopy in December but showed the highest percentage in March, which is statistically equal to the field estimations. The SVM classification accounted for the lowest value in the February measurement but the highest value occurred during May. The OORF classification, compared to the other methods, recorded the highest values for canopy damage at the beginning and at the end of the study cycle. However, the most noticeable aspect is that this supervised classification methodology showed statistically equal results as those of the SVM, which followed an analogous supervised procedure throughout the complete study period.

According to the results exhibited in Fig. 3, and given the same trend shown by the four implemented methods and the number of burrows, the correlation between the percentage of damage and the number of burrows m^−2^ was explored (Table 3). The resulting correlations were all statistically significant (P<0.01), with the SVM classification having the highest coefficient value, closely followed by the NDVI segmentation, with the OORF classification in third place. The ISODATA classification showed the lowest correlation with the number of burrows. Likewise, the correlation between the field estimates of the % damaged areas and the classified maps, all of them significant, showed the highest values for the SVM method and the lowest for ISODATA.

**Table 3:** Pearson linear correlation coefficients between the remote methodologies and the number of active burrows

	NDVI segmentation	Supervised pixel‐based classification (SVM)	Unsupervised pixel‐based classification (ISODATA)	Supervised object‐based classification (OORF)
#Burrows	0.534*	0.577*	0.256*	0.439*
Field Est.	0.455*	0.513*	0.300*	0.376*

* Correlation significant at the *P <* 0.01 level (two‐tailed). #Burrows, number of burrows m^−2^.

NDVI, normalized difference vegetation index; SVM, support vector machine; ISODATA, iterative self‐organizing data analysis; OORF, object‐oriented random forest; Field Est., field estimations.

## 4 DISCUSSION

Remotely sensed multispectral imagery is a common method for evaluating the status of the crop canopy by means of vegetation indices or multispectral classifications.^42^ In this situation, drones were the preferred tool for imagery acquisition because of the small size of the agricultural plots and the very high resolution needed to achieve an accurate estimation of the damage.

The four classification methods exhibited similar performances in terms of the percentage of overall accuracy and false negatives. Nevertheless, following the recommendations of Anderson *et al*.^43^ and Thomlinson *et al*.,^44^ only the NDVI segmentation and the OORF classification maps had a sufficiently low degree of uncertainty, although the limits for the minimum level of overall accuracy are quite variable in the literature. Interestingly, if the accuracy is analyzed date‐by‐date, we found that the maps with the lowest accuracy corresponded to the February flight, coinciding with the highest canopy damage. Therefore, although the percentage of damaged areas should be analyzed carefully, the most accurate results belonged to the NDVI segmentation. Notably, simpler methods, such as the unsupervised classification provided acceptable results while avoiding subjectivity in the clustering algorithm. This is particularly interesting when trying to translate these geotechnologies into real‐world field monitoring results. Indeed, the introduction of new geotechnologies holds the promise of increasing both agricultural productivity and the welfare of farmers.^45^ However, it is prudent to point out that unsupervised methods perform better when assigning broad or uncomplicated classes, such as ‘bare ground’ *versus* ‘vegetated cover’,^46^ as was done in this study with ‘damaged’ *versus* ‘nondamaged’. Categories with less separability may benefit from a supervised approach. In addition, the OORF method achieved slightly better results than the pixel‐based methods, indicating that object‐oriented classifications are less influenced by the salt‐and‐pepper effect^47^ *–* the distribution of speckled pixels between different classes^48^ – which could artefact the classification results. The OORF method's elevated cost in training and computing outweighs this modest improvement, especially in the context of moderately skilled users.

It seems that implementing simple methods that require little technical training but are useful for farmers to control their crop status should be compulsory. These results reveal a clear relationship between vole activity (number of burrows m^−2^) and the observed damage to the alfalfa cover. Specifically, the greatest canopy damage, which was observed in February 2021, implied a larger damaged area than in March 2021 (Table 3). Hence, it could be assumed that there is a significant correlation between the vole damage to the alfalfa canopy and the number of burrows, althought this relation should be verified with distributions other than a linear one. This significant relationship was in agreement with the correlation found between the number of burrows and the field‐observed damaged areas, with a Pearson linear coefficient of 0.591^**^.

In some agricultural areas of the Czech Republic, Truszkowski^49^ reported that with moderate populations of voles (129 voles ha^−1^), ≈198 kg of alfalfa dry mass ha^−1^ are consumed, but during population outbreaks (551 voles ha^−1^), they can consume almost one ton of alfalfa dry mass ha^−1^. Others suggest that a population density of 200 voles ha^−1^ can consume ≥5% of the alfalfa plants,^50^ which is economically important. However, the problem is related not only to the aboveground consumed biomass, but also to the total biomass eliminated (cut down) for further production; the latter is greater than the former by almost a factor of four.^49^

Active burrow counts would be useful if they varied consistently with the fluctuations in the rodent population density.^51^ In our work, a significant relationship between vole activity (and thus the vole population), expressed as the number of burrows m^−2^, and the observed damage to the alfalfa canopy was found on the measurement dates when field estimations were available (February and March). However, it was even more evident in February, where all four classification methods and the field estimations reached their peak values, coinciding with a density of 1.33 burrows m^−2^.

It is worth mentioning the relationship observed between the resulting population dynamics of the vole and meteorological conditions during the study period. A temperature anomaly took place in January 2021, with extremely low values (−11 °C), making it the coldest January since 1985.^52^ Two difficult cold periods occurred in January 2021. The first occurred between the 5th and 8th, and the second cold period extended from the 11th to 18th. The latter was exceptionally intense and began after the passage of the Filomena storm and the establishment of an anticyclone over the peninsula. This phenomenon resulted in heavy snows covering large regions, which led to unusually low temperatures, reaching values below −20 °C in some areas.^52^

There was a sharp decline in the vole population, which resulted in increased canopy coverage from February until the end of the studied period. We believe that these cold temperatures might have negatively affected the fecundity of voles and the ensuing canopy recovery. Related to this fact, Giraudoux *et al*.^3^ found a strong but complex relationship that proved that female vole reproduction was depressed after cold winters.

## 5 CONCLUSION

The change in agricultural practices in recent years, with the introduction of new cropping systems and new technologies, together with the influence of external factors such as climate change and natural resource conservation policies, makes it imperative to design new approaches to control troublesome species, such as voles, that cause pests and diseases. Voles have drawn the attention of not only those involved in the agricultural sector, but also of society as a whole as a consequence of the impact that they have had on the economy, food safety, health and environmental protection. In this research, new UAS‐based alternatives were used to monitor and estimate vole damage in an agricultural field. A multispectral camera onboard the drone provided aerial imagery to test four classification methods. All of them showed similar performances, although the NDVI segmentation exhibited the most accurate and reliable results. Moreover, a significant relationship between vole activity (measured by the number of burrows in active colonies) and observed damage to the alfalfa canopy was identified.

Unmanned aerial systems and multispectral imagery classification proved to be an effective and easily transferable methodology to help identify vole damage to the alfalfa field and to inform integrated management programs against this pest. The combination of these techniques with field measurements of vole activity, such as the number of active burrows, revealed the actual fluctuations of the common vole population. These variations, specifically the sharp decrease in vole activity and alfalfa damage up to the beginning of March, were coincidental with a strong negative temperature anomaly that occurred in January 2021.

In the foreseeable future, the applicability of this method can be further extended by expanding the automation of burrow counting by object detection algorithms from the deep learning domain.

## CONFLICT OF INTEREST

The authors declare that they have no conflicts of interest.

## Data Availability

The data that support the findings of this study are available from the corresponding author upon reasonable request.
